# Scale Design of Dual-Layer Polyphenylsulfone/Sulfonated Polyphenylsulfone Hollow Fiber Membranes for Nanofiltration

**DOI:** 10.3390/membranes13080714

**Published:** 2023-08-01

**Authors:** Javed Alam, Arun Kumar Shukla, Lawrence Arockiasamy, Mansour Alhoshan

**Affiliations:** 1 King Abdullah Institute for Nanotechnology, King Saud University, P.O. Box 2455, Riyadh 11451, Saudi Arabia; ashukla@ksu.edu.sa (A.K.S.); ldass@ksu.edu.sa (L.A.); mhoshan@ksu.edu.sa (M.A.); 2 Department of Chemical Engineering, College of Engineering, King Saud University, P.O. Box 2455, Riyadh 11451, Saudi Arabia

**Keywords:** hollow-fiber membranes, dual-layer membranes, nanocomposite membranes, selective separation

## Abstract

This study focuses on the synthesis and characterization of dual-layer sulfonated polyphenylenesulfone (SPPSu) nanocomposite hollow fiber nanofiltration membranes incorporating titanium dioxide (TiO_2_) nanoparticles through the phase inversion technique. Advanced tools and methods were employed to systematically evaluate the properties and performance of the newly developed membranes. The investigation primarily centered on the impact of TiO_2_ addition in the SPPSu inner layer on pure water permeability and salt rejection. The nanocomposite membranes exhibited a remarkable three-fold increase in pure water permeability, achieving a flux of 5.4 L/m^2^h.bar compared to pristine membranes. The addition of TiO_2_ also enhanced the mechanical properties, with an expected tensile strength increase from 2.4 to 3.9 MPa. An evaluation of salt rejection performance using a laboratory-scale filtration setup revealed a maximal rejection of 95% for Mg_2_SO_4_, indicating the effective separation capabilities of the modified dual-layer hollow fiber nanocomposite membranes for divalent ions. The successful synthesis and characterization of these membranes highlight their potential for nanofiltration processes, specifically in selectively separating divalent ions from aqueous solutions, owing to their improved pure water flux, mechanical strength, and salt rejection performance.

## 1. Introduction

Water scarcity has become a critical global issue impacting numerous regions worldwide. Factors such as population growth, urbanization, and climate change have exacerbated the demand for clean and fresh water, while the availability of freshwater resources continues to diminish. This escalating water scarcity necessitates innovative solutions to address the challenge and ensure sustainable water management practices [1,2,3,4].

Membrane technology has emerged as a crucial tool in tackling water scarcity by offering efficient and effective water treatment and purification solutions. Membrane-based processes provide several advantages, including high efficiency, low energy consumption, and the ability to remove contaminants at the molecular level [3]. These processes find extensive application in desalination, wastewater treatment, and water reuse. Among the various membrane configurations, hollow-fiber membranes have gained significant attention in academia and industry due to their inherent advantages, including a large surface area per unit volume, favorable mechanical strength, and ease of handling. However, the performance of conventional hollow-fiber membranes requires further improvement to achieve highly selective separation while maintaining enhanced thermal and mechanical stability [5,6,7,8,9,10,11,12].

In response to these challenges, researchers have directed their focus towards the development of dual-layer hollow-fiber (DLHF) membranes, which offer the potential for a selective surface layer with a mixed matrix. This unique characteristic enables highly selective separation while maintaining excellent mechanical strength, allowing for long-term operation without fiber failure or significant deterioration. Recent studies have demonstrated successful applications of DLHF membranes in various separation processes [13,14,15]. The fabrication of dual-layer hollow fiber membranes has garnered considerable interest due to its numerous advantages, including low material cost, favorable thermal and mechanical stability, and the ability to optimize membrane performance using high-performance selective layers [12,16,17,18]. Notable advancements in this field include the work of Liu et al. [15], who reported the fabrication of a dual-layer hollow-fiber membrane using poly(styrene-b-4-vinylpyridine)-based (PS4VP-based) and poly(vinylidene fluoride)-based (PVDF-based) materials. The membrane was fabricated using block copolymer self-assembly through non-solvent induced phase separation, resulting in a highly ordered isoporous surface and excellent mechanical strength. Furthermore, dual-layer nanocomposite hollow fiber membranes composed of polyethersulfone (PES) and PVDF, incorporating multi-walled carbon nanotubes (MWCNTs), have been successfully fabricated using a two-step thermally induced phase separation/non-solvent induced phase separation (TIPS/NIPS) process. These membranes exhibited the ability to withstand high pressures with relatively low membrane compaction, making them suitable for ultrafiltration applications. The fabrication of novel dual-layer hollow-fiber membranes using a single-step co-extrusion technique has also been explored by Dzinun et al. [19]. These membranes incorporated immobilized titanium dioxide (TiO_2_) nanoparticles in their outer layer, exhibiting favorable interfacial adhesion and well-dispersed TiO_2_ particles. Comparative studies revealed the superior mechanical strength of DLHF membranes compared to flat sheet cellulose acetate (CA) membranes, with smaller pore sizes in the outer layer of the hollow-fiber membranes. Additionally, dual-layer hollow-fiber membranes based on polybenzimidazole (PBI) and PES materials have demonstrated high rejection rates for heavy metals such as cadmium, lead, and chromium, as reported by Zhu et al. [14]. These advancements highlight the progress made in the field of dual-layer hollow fiber membranes, showcasing the potential for enhanced membrane performance and the utilization of various functional materials to achieve specific separation requirements.

Polyphenylsulfone (PPSu) stands out as an especially promising membrane material for ultrafiltration, nanofiltration, reverse osmosis, and forward osmosis. PPSu possesses desirable characteristics, including ease of membrane formation and favorable thermal and mechanical stability, making it an ideal choice for membrane materials [20]. Moreover, PPSu exhibits superior properties compared to commonly used materials like polysulfone (PSf) and polyether sulfone (PES). Furthermore, the relatively low cost of PPSu resin enhances its appeal as an alternative for next-generation membrane preparation. However, similar to PSf and PES, PPSu is prone to fouling due to its hydrophobic nature. Fouling not only increases the frequency of membrane cleaning and energy expenditure required to maintain productivity but also necessitates larger membrane areas and frequent membrane replacements. Therefore, alternative approaches must be explored to develop more hydrophilic membranes through chemical modification, surface membrane functionalization, and incorporation of nanoparticles within the membrane matrix (MMM) [21,22,23,24]. The incorporation of different nanomaterials in polymer matrices has gained significant attention due to their ability to impart unique properties to the resulting polymer nanocomposites. Various types of nanomaterials have been explored for their incorporation, including carbon-based nanomaterials such as carbon nanotubes (CNTs) and graphene, metallic nanoparticles such as silver (Ag) and gold (Au), metal oxide nanoparticles such as titanium dioxide (TiO_2_) and zinc oxide (ZnO), and clay nanoparticles like montmorillonite (MMT) and halloysite nanotubes (HNTs). Each of these nanomaterials offers distinct characteristics that can be beneficially transferred to the polymer matrix [25,26,27,28,29,30,31].

The incorporation of TiO_2_ nanoparticles into polymer matrices has gained significant attention in recent years due to their potential for synergistic effects in various applications. In particular, the combination of TiO_2_ nanoparticles with SPPSu polymer presents a promising strategy for improving the performance of membranes. The synergy between TiO_2_ nanoparticles and SPPSu polymer arises from their complementary properties and functionalities. TiO_2_ nanoparticles offer advantages such as high surface area, photocatalytic activity, and enhanced mechanical strength, while SPPSu polymer provides excellent ion exchange capacity and thermal stability. By combining these two components, it is possible to exploit their individual strengths and achieve enhanced membrane properties, including improved water permeability, selectivity, fouling resistance, and stability. This introduction aims to provide an overview of the potential synergistic effects resulting from the combination of TiO_2_ nanoparticles with SPPSu polymer and highlights the importance of understanding their interactions and optimized integration in membrane systems. Various researchers have presented SEM, EDX, FTIR, and XRD analyses of well-known TiO_2_ nanoparticles [32,33,34].

The main objective of this work is to design and fabricate dual-layer sulfonated polyphenylsulfone nanocomposite hollow fiber nanofiltration membranes for high-efficiency salt separation. By incorporating innovative strategies such as chemical modification, surface membrane functionalization, and nanoparticle loading within the inner surface of membrane matrix, these membranes aim to enhance their hydrophilicity, surface charge, surface morphology, and achieve high-efficiency salt separation. The purpose of choosing a TiO_2_ incorporated PPSu/SPPSu dual-layer structure in our study is to capitalize on the unique properties and synergistic benefits offered by these two materials. Firstly, PPSu is known for its excellent mechanical strength, thermal stability, and chemical resistance. It is widely used in various applications where robustness and durability are crucial factors. By incorporating PPSu as one of the layers in our dual-layer structure, we aim to enhance the overall mechanical integrity and long-term stability of the membrane. On the other hand, SPPSu is a sulfonated variant of PPSu, which possesses ion-exchange properties due to the presence of sulfonic acid groups. These ion-exchange characteristics make SPPSu suitable for various applications in the field of membrane technology, particularly in water treatment. By utilizing SPPSu as the second layer in our dual-layer structure, we introduce the potential for ion selectivity and facilitated transport of specific ions or molecules through the membrane. The combination of PPSu and SPPSu in a dual-layer structure allows us to harness the strength and stability of PPSu while incorporating the ion-exchange properties of SPPSu. This design strategy aims to optimize the membrane’s performance by achieving a balance between mechanical robustness and ion selectivity. Furthermore, the dual-layer structure provides additional advantages such as the prevention of delamination between layers, improved adhesion, and enhanced overall membrane integrity.

## 2. Materials and Methods

### 2.1. Materials

Polyphenylsulfone (PPSu) was kindly supplied by Solvay Advanced Polymers, Milano, Italy, and served as the main polymer matrix for the fabrication of the membranes. Polyethylene glycol-600, magnesium sulfate, sodium sulfate, sodium hydroxide, potassium chloride, sodium azide, and glycerol were obtained from Merck, Rahway, NJ, USA and used as received. Chlorosulfonic acid, an important reagent in the synthesis of sulfonated polyphenylenesulfone (SPPSu), was also obtained from Merck, Rahway, NJ, USA. Titanium dioxide nanoparticles, which played a crucial role in enhancing the properties of the membranes, were obtained from Sigma–Aldrich, Rahway, NJ, USA. Sodium dodecyl sulfate (SDS), a surfactant, was also obtained from Sigma–Aldrich and used in the membrane preparation process. N-Methyl Pyrrolidone and dichloromethane were sourced from SD Fine-Chem Limited, Tamil Nadu, Chennai, India, and used as solvents in the synthesis and processing of the membranes. Millipore MQ purified water was used for the experiments and to prepare the gelation bath, ensuring the purity of the water used in the membrane fabrication process.

### 2.2. Synthesis of Sulfonated Polyphenylsulfone

The synthesis of sulfonated polyphenylsulfone (SPPSu) was conducted using a procedure reported in the literature, involving the use of chlorosulfonic acid. Initially, predried PPSu was dissolved in dichloromethane (DCM) by constant stirring. The chloro sulfonic acid was slowly added to the DCM solution over a period of 2 h at 0 °C using a dropping funnel. The resulting solution, which became viscous, was further stirred for 2 h at room temperature. To precipitate the SPPSu, the viscous polymer solution was treated with ethanol, and the resulting precipitate was thoroughly washed until the pH reached 7. The obtained white precipitate of SPPSu was then dried for 24 h in a vacuum oven. To modify the SPPSu, it was re-dissolved in NMP, and an excess amount of sodium methoxide was added. The mixture was continuously stirred for 3 h to allow for the reaction to take place. The sodium salt of SPPSu was then re-precipitated in distilled water and subsequently dried in a vacuum oven. The resulting dried SPPSu exhibited a highly crystalline structure, making it suitable for the preparation of hollow-fiber membranes and blend nanocomposite hollow-fiber membranes [35]. This synthesized sulfonated polyphenylenesulfone offers additional properties beyond those of the polyphenylenesulfone polymer. The highly crystalline nature of the SPPSu ensured its stability and enhanced performance in subsequent membrane fabrication processes. The incorporation of additional sulfone functional groups into the PPSU polymer matrix expands its potential applications and facilitates further customization of the resulting membranes, enabling tailoring to specific requirements. Overall, this synthesis process laid the foundation for the preparation of hollow-fiber membranes and blend nanocomposite hollow-fiber membranes with improved properties and performance.

### 2.3. Fabrication of DLHF Nanocomposite Membrane and Module

The fabrication process of the dual-layer hollow-fiber nanocomposite membrane and module is described in detail as follows.

First, PPSu, SPPSu, and TiO_2_ nanopowder were dried overnight at 60 °C in a vacuum oven to eliminate moisture content. The concentrations of the dope solutions were determined based on the viscosity–concentration profile. A 24 wt% PPSu solution was chosen as the outer dope solution, while a 22 wt% SPPSu solution was used as the inner dope solution [36]. For the inner dope solution, TiO_2_ was dispersed in NMP and sonicated for 40 min using a Branson digital probe sonicator. After sonication, the TiO_2_ dispersion was mixed with the SPPSu polymer solution. The outer dope solution was prepared by dissolving PPSu polymer in NMP solvent and subsequently incorporating PEG-600 directly into the solution. The specific blend compositions are provided in Table 1. Both mixtures were stirred at 70 °C for 24 h to achieve a homogeneous solution and subsequently degassed in a sealed container for 12 h to remove any trapped air bubbles.

During the spinning process, a bore fluid composed of a 9:1 ratio of double distilled water and NMP was used. Tap water served as the external coagulant. Hollow-fiber membranes were prepared using a hollow fiber spinning line from Delta, Via Pietro Bucci, Cubo, Italy. The spinning process involved employing triple-orifice spinnerets and a spinning machine, and spinning conditions of dual-layer hollow-fiber membranes provided in Table 2. The spun hollow-fiber membranes were then immersed in a water bath for 24 h to remove residual solvent. To improve membrane wettability and prevent pore collapse, the membranes underwent post-treatment using a 20% glycerol solution for 1 h. Following post-treatment, the membranes were dried at room temperature for 72 h before module fabrication.

For module preparation, defect-free hollow fibers (100 in total) were selected. These hollow fibers were wrapped with a polymeric mesh and inserted into acrylic-based modules. The ends of the module were sealed using epoxy resin. Freshly prepared epoxy resin was poured onto the module through the permeate pathway. The module was shaken thoroughly and then dried.

### 2.4. Rheological Properties of Dope Solution

The rheological properties of the dope solution were evaluated using an MCR300 rheometer from Anton Parr, Graz (Austria). The measurements were conducted with a PP50 measuring plate with a diameter of 50 mm and a measuring position gap of 1 mm. The angular frequency (ω) of the rheometer was set in the range of 0.01 to 100 rad/s to obtain the necessary rheological parameters and viscosity values. All experiments were conducted under a nitrogen atmosphere to prevent any unwanted reactions or interactions.

### 2.5. Membrane Characterization

#### 2.5.1. Morphological Studies

The cross-sectional morphology of the prepared DLHF membrane was analyzed using a JEOL instrument from Akishima, Tokyo, Japan through Scanning Electron Microscopy (SEM). Prior to imaging, the membrane samples were allowed to air dry to remove any surface water. The dried samples were then fractured under cryogenic conditions using liquid nitrogen and further dried at a temperature of 21 °C. To enhance the electrical conductivity of the polymeric membranes for SEM imaging, a thin layer of gold was sputtered onto the sample surface. The SEM images were captured under a high vacuum environment at an operating voltage of 5–15 kV, depending on the specific characteristics of the sample being analyzed. This allowed for detailed visualization and examination of the cross-sectional structure of the dual-layer nanocomposite hollow fibers. The SEM images provided insights into the internal morphology, layer arrangement, and overall integrity of the membranes.

Furthermore, Atomic Force Microscopy (AFM), Bruker, San Jose, CA, USA, was employed to characterize the surface features of the membranes, such as texture and roughness. Surface imaging was conducted in tapping mode at ambient temperature using a silicon (Si) tip. The AFM measurements were performed with a scan frequency of 0.999 Hz using the 6.13 Veeco NanoScope V MultiMode software. The scan ranges for surface roughness and structure analysis were set at 5 × 5 μm and 500 × 500 nm, respectively. By utilizing AFM, the topography and surface properties of the DLHF could be examined at a nanoscale level, providing information about the surface roughness, texture, and any potential surface modifications or enhancements.

The surface elemental compositions of the membranes were quantified using X-ray photoelectron spectroscopy (XPS) with a JEOL JPS-9030 instrument from Akishima, Tokyo, Japan. For the analysis of nanocomposite membrane samples, X-ray diffraction (XRD) patterns were obtained using an Ultima IV X-ray diffractometer (Akishima, Tokyo, Japan) with high-intensity Cu-Kα radiation. The measurements were performed on oriented samples, scanning over an angle range from 10° to 60°.

#### 2.5.2. Measurement of Membrane Zeta Potential

The zeta potential of a manufactured hollow fiber (HF) membrane (inner surface) was measured using the tangential streaming potential method using a SurPASS” electrokinetic analyzer (Anton Paar, GmbH, Graz, Austria). The membrane sample was preserved to a length of 5 mm and put into a sample holder of HF membrane with a diameter of 1.4 mm. The zeta potential of the inner surface of a hollow fiber membrane as a function of pH was calculated using a 1 mM KCl electrolyte solution. SurPASS’s in-built titration device has been used to test the streaming potential under various pH conditions ranging from pH 6 to pH 2. In addition, the pH of the electrolyte solution was adjusted using a titration device and 0.05 M HCl and 0.05 M NaOH. Finally, Attract^®^ software estimated zeta potential from streaming potential readings using the Fairbrother and Mastin (F-M) equation provided in Equation (1):

(1) $$\zeta =\frac{\Delta U}{\Delta p}\times \frac{\eta }{\varepsilon \times \varepsilon_{0}}\times \frac{L}{D}$$

where Δ*U* and Δ*P* are streaming potential (mV), applied pressure (Pa), and *L* and *D* are the channel length (m) as well cross section area (m^2^), respectively.

#### 2.5.3. Porosity and Contact Angle Measurement

The porosity of the hollow fibers is determined by soaking the membranes in kerosene for 2 days. The residual kerosene on the surface of the hollow fibers will be removed by blotting with tissue paper. The mass of the membranes before and after immersing in kerosene will be obtained using a digital microbalance. The porosity of the membranes (ε) is defined as the pore volume divided by the total volume of the membrane as follows:

(2) $$\varepsilon \left(\%\right)=\frac{W_{W}-W_{D}}{\rho \;.\;A\;.\;l_{m}}\times 100\;$$

where $W_{W}$ represents the weight of the wet membrane, $W_{D\;}$ represents the weight of the dry membrane, A represents the membrane effective area (m^2^), ρ represents the water density (0.998 gcm^−3^), and $l_{m}$ represents the membrane thickness (m).

The contact angle of the hollow-fiber membranes was measured by the sessile drop technique. Ten different locations had their contact angles measured, and the average results were then given. The drop-shape analysis system was entirely automated, and the contact angles were measured.

#### 2.5.4. Thermal and Mechanical Properties of the Membranes

A Thermogravimetric analyzer (Giessen, Germany, METTLER) was used to monitor the degradation temperature while heating the manufactured hollow-fiber membranes at a rate of 10 °C min^−1^ in order to examine their thermal stability.

The Double column Lloyd (UTM 5KN Model LR5K Plus, USA) was used to test the mechanical parameters of the hollow fibers at room temperature, including extension at break, tensile strain, and the Young’s modulus. In order to obtain reliable findings, at least five membrane samples per membrane were analyzed.

#### 2.5.5. Performances of the Membranes

The pure water flux through the membranes will be determined by measuring the time required for the permeation of a specific amount of water. The flux of water will be calculated using the following formula:

(3) $$J_{w}=\frac{V}{At}=\frac{Q}{A∆t}$$

where J_w_ is the water flux (L/m^2^ h); Q is the quantity of water permeated (l); Δt is the sampling time (h); and A is the membrane area (m^2^).

Water permeability (W_p_) was determined by calculating the slope of the linear relationship between the water flux and the transmembrane pressure (∆P), as described by the following formula:

(4) $$W_{p}=\frac{J_{w}}{∆P}$$

The rejection performances of the membrane module were evaluated using a cross-flow system operating in total recycle mode. Two distinct salt solutions, Na_2_SO_4_ and MgSO_4_, were prepared with an identical concentration of 500 ppm. The experiments were conducted at a pressure of 3 bar and neutral pH conditions. Solute rejection was determined by calculating the ratio between the permeate concentration (Cp) and the feed concentration (Cf) using Equation (5). The concentrations of the feed and permeate samples were analyzed using Ion Chromatography (ICS 5000-DIONEX, Ramsey, MN, USA) to determine the rejection ability of the membrane module.

(5) $$Rejection\%=1-\frac{Cp}{Cf}\times 100$$

All experiments were conducted three times to minimize errors, and the average values were calculated.

In order to ascertain the molecular weight cutoff (MWCO) of the membrane, we utilized a PEG solution with a concentration of 1.0 g/L. More specifically, PEG with different molecular weights spanning from 200 to 1000 Da was selected for the experiments. The concentrations of PEG present in both the feed and permeate solutions were determined using a Sievers 5310 C total organic carbon analyzer, which is manufactured by GE Analytical Instruments, Boston, MA, USA. Subsequently, the rejection of the PEG solution was calculated using Equation (4).

## 3. Results and Discussion

### 3.1. Rheological Properties of the Dope Solution

**Figure 1:**
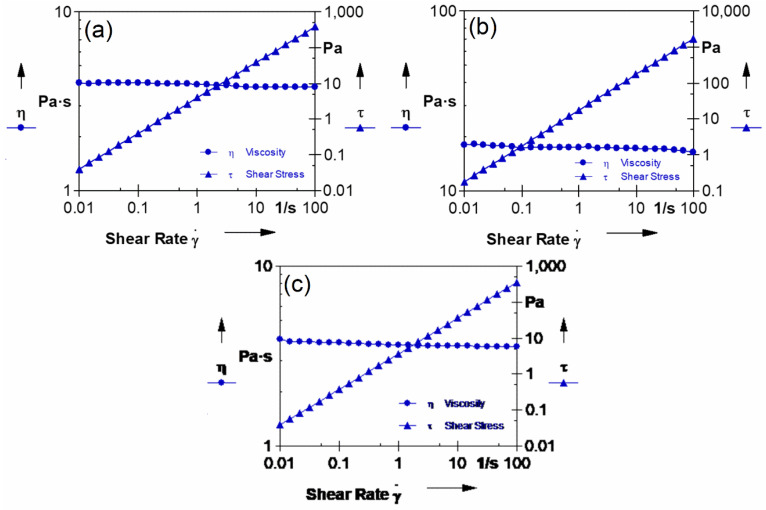
Rheological properties of the (**a**) PPSu (**b**) SPPSu, and (**c**) SPPSu-TiO_2_ (1.0 wt%) dope solution.

The high viscosity of the solution indicates that the overall diffusion between components in the phase-inversion system is hindered kinetically. This can be attributed to an increase in rheological hindrance or a delayed exchange between the solvent and non-solvent, which ultimately affects the characteristics of the resulting membranes. Therefore, conducting rheological studies on the dope solution is crucial for enhancing membrane performance. In order to determine the inner and outer dope solution viscosity at the hollow-fiber spinning temperature of 50 °C, the viscosity was measured as a function of shear rate. The results of this study are presented in Figure 1. The significant increase in viscosity observed in the SPPSu-TiO_2_ (1.0 wt%) dope solution compared to the pure SPPSu solutions highlights the influence of TiO_2_ nanoparticles on the rheological properties of the polymer solution. This viscosity enhancement can be attributed to the interactions between the nanoparticles and the polymer matrix, leading to an increase in effective polymer chain length and entanglement density. The presence of TiO_2_ nanoparticles also introduces a hindrance effect on the flow of the solution, resulting in higher resistance and elevated viscosity values. Moreover, the nanoparticles can affect solvent–polymer interactions, influencing the segmental mobility and intermolecular associations within the solution. These combined factors contribute to the observed significant increase in viscosity and emphasize the complex interplay between the nanoparticles, polymer matrix, and solvent in determining the rheological behavior of the SPPSu-TiO_2_ (1.0 wt%) dope solution. This can be attributed to the addition of TiO_2_ nanoparticles in the solution. Overall, these findings emphasize the importance of understanding and controlling the rheological properties of the dope solution, as it directly impacts the performance of the resulting membranes.

### 3.2. Membrane Morphology

**Figure 2:**
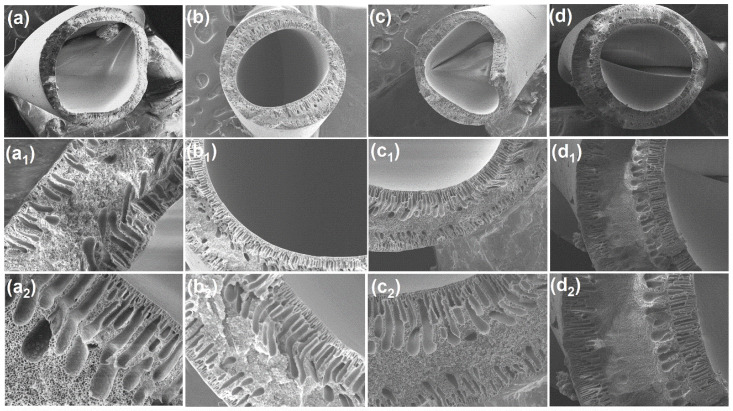
SEM cross-sectional images with high magnifications of (**a**) SPPSu/PPSu, (**b**) SPPSu-TiO_2 (0.50 wt%)_/PPSu, (**c**) SPPSu-TiO_2 (1.0 wt%)_/PPSu, and (**d**) SPPSu-TiO_2 (2.0 wt%)_/PPSu membranes, a1 and a2; b1 and b2; c1 and c2; d1 and d2 are images of different zoom level of a, b, c, d, respectively.

In this study, Figure 2a–d presents the SEM images illustrating the cross section of the DLHF membranes that were prepared. The observation of delamination between the two layers in the material is particularly interesting. This phenomenon can be explained by the fact that both the inner and outer dope solutions were synthesized using the same material. Delamination refers to the separation or detachment of layers within a material, and in this case, it occurred between the inner and outer layers. When the same material is used to synthesize both the inner and outer dope solutions, it can lead to a lack of interfacial compatibility between the layers. The resulting mismatch in properties or molecular structure could create stress or weak points at the interface, making it susceptible to delamination. This finding highlights the importance of carefully considering the selection and compatibility of materials when designing multi-layered structures to avoid delamination issues. Despite the delamination, the cross section morphology of the hollow fibers exhibited a moderately symmetric structure in both the inner and outer layers of the membrane. The morphology of the hollow fibers is influenced by the concentrations of polymer dope and the air gap employed in the fabrication process. In this investigation, a constant air gap of 10 cm was maintained, indicating that the primary factor impacting the morphology is the polymer concentration in the starting dope solution. Detailed information regarding the specific concentrations used can be found in Table 1 of the manuscript. Regarding the addition of different concentrations of TiO_2_ into the SPPSu polymer matrix in the inner layer, the images clearly demonstrate that this addition resulted in significant structural changes in the cross section of the membranes. The morphology of the DLHF nanocomposite membrane reveals a very thin top layer with nanopores. On the other hand, the incorporation of PEG 600 on the outer surface of PPSu accelerated the demixing of the solvent and non-solvent, leading to the formation of microvoids in the cross section of the outer layer [27,37]. Importantly, the cross-section SEM morphology images of the produced membranes were found to be consistent with the water permeability results obtained, establishing a clear correlation between the observed structural features and the transport properties of the membranes. This alignment between the SEM images and the water permeability data reinforces the understanding that the membrane’s internal structure directly influences its ability to facilitate or restrict the passage of water molecules. The visual evidence provided by the SEM analysis strengthens the validity of our findings, highlighting the significance of the observed structural characteristics in determining the membrane’s transport behavior.

**Figure 3:**
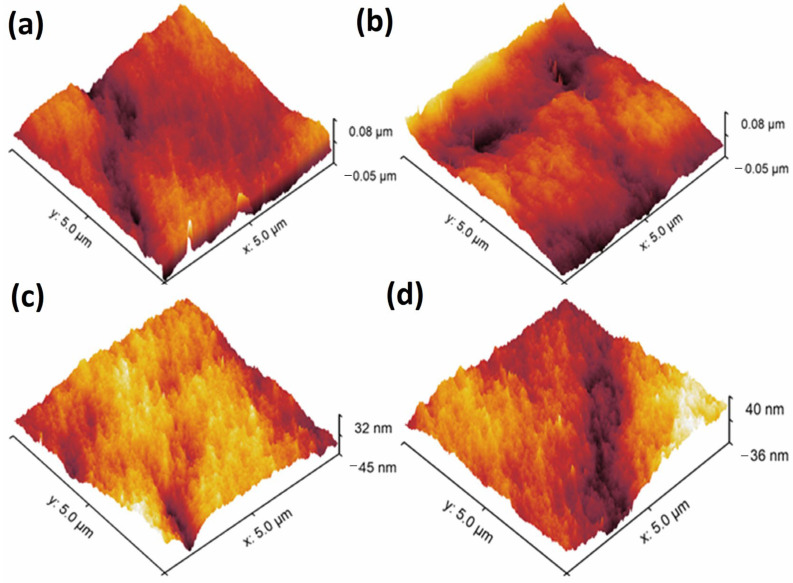
AFM 3D images belongs to DLHF membranes inner surface of (**a**) SPPSu/PPSu, (**b**) SPPSu-TiO_2 (0.50 wt%)_/PPSu, (**c**) SPPSu-TiO_2 (1.0 wt%)_/PPSu, and (**d**) SPPSu-TiO_2 (2.0 wt%)_/PPSu).

Moreover, the inner layer surface roughness of the prepared DLHF membranes was quantified using AFM analysis. Figure 3a–d presents the AFM 3D images and average roughness values (Ra). The authors reported that the surface roughness of the SPPSu/PPSu membrane, without the addition of TiO_2_, was measured to be approximately 8 nm. However, upon incorporating different concentrations of TiO_2_ nanoparticles into the SPPSu matrix in the inner surface, the roughness of the membranes increased significantly. Specifically, the roughness values were measured as 12 nm, 15 nm, and 21 nm for SPPSu-TiO_2_ (0.50 wt%)/PPSu, SPPSu-TiO_2_ (1.0 wt%)/PPSu, and SPPSu-TiO_2_ (2.0 wt%)/PPSu, respectively. The observed increase in surface roughness can be attributed to the incorporation of hydrophilic groups into the SPPSu polymer matrix. These hydrophilic moieties promote the sudden demixing of the solvent and water during the phase inversion process. Consequently, a greater degree of phase separation occurs, resulting in the formation of a rougher surface in the inner layer of the DLHF membranes.

**Figure 4:**
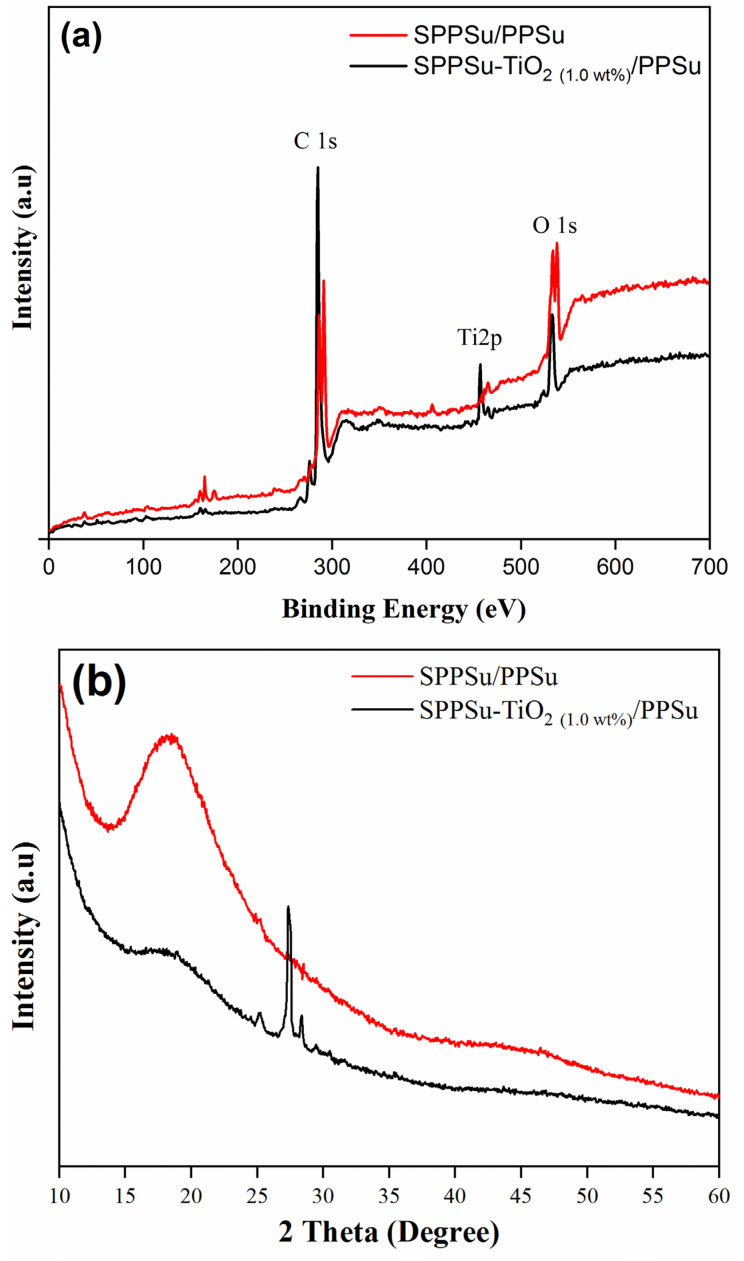
Spectral analysis of DLHF membranes inner surface. (**a**) X-ray photoelectron spectroscopy, (**b**) X-ray diffraction.

The chemical composition analysis of the SPPSu/PPSu and SPPSu-TiO_2 (1.0 wt%)_/PPSu nanocomposite DLHF membranes was conducted using XPS. The XPS spectra provided valuable information about the main elemental peaks on the membrane surface. Specifically, the oxygen (O 1 s) and carbon (C 1 s) peaks were observed at 535 and 287 eV, respectively (Figure 4a). Upon incorporating TiO_2_ nanomaterials into the SPPSu/PPSu matrix, an additional distinct Ti2p peak was observed at 457 eV, confirming the successful attachment of TiO_2_ onto the polymer matrix.

Furthermore, to gain more insight into the structure of the nanocomposite membranes, XRD patterns were obtained (Figure 4b). The XRD spectra revealed the crystalline nature of the TiO_2_ nanomaterials, further supporting their effective incorporation into the polymer matrix. The crystallinity of the nanocomposite membrane is a critical factor influencing its transport properties. In the XRD pattern of the SPPSu-TiO_2 (1.0 wt%)_/PPSu nanocomposite, two major characteristic peaks were identified. The broad peak at 2θ = 19° was attributed to the SPPSu polymer, while the crystalline peaks at 2θ = 27° corresponded to the presence of TiO_2_. Importantly, the results indicated that the addition of TiO_2_ into the polymer matrix did not lead to significant changes in the amorphous structure of the SPPSu-TiO_2 (1.0 wt%)_/PPSu nanocomposite DLHF membrane.

### 3.3. Porosity, Hydrophilicity and MWCO of Membrane

**Figure 5:**
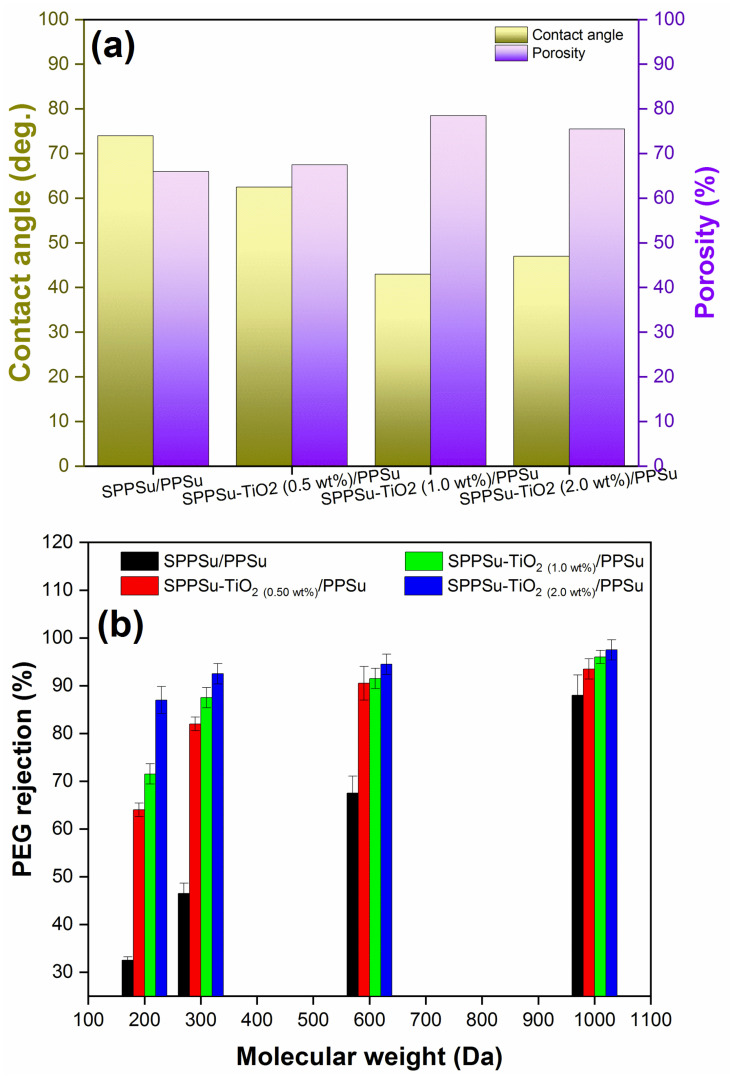
Prepared DLHF membranes’ (**a**) water contact angle and porosity, (**b**) molecular weight cutoff.

The performance of membranes is greatly influenced by their surface properties, particularly porosity and hydrophilicity. In a study, these properties were investigated using overall porosity and contact angle measurements, and the results are presented in Figure 5. When examining the overall porosity, it was observed that the inclusion of TiO_2_ nanoparticles in the SPPSu/PPSu DLHF nanocomposite membranes led to a significant increase in porosity compared to the pure SPPSu/PPSu membrane. The addition of TiO_2_ resulted in an increase in the void volume within the substructure of the fibers, as evidenced by morphological studies. The presence of TiO_2_ nanoparticles in the membrane formulation appears to play a significant role in the formation of a more porous membrane structure. The incorporation of TiO_2_ nanoparticles likely influences the phase separation process during membrane fabrication, leading to increased pore formation. TiO_2_ nanoparticles can act as nucleation sites for pore formation, facilitating the creation of void spaces within the membrane matrix. Additionally, the interaction between the nanoparticles and the polymer matrix can induce phase separation and enhance the demixing of the polymer solution, further promoting the development of a porous structure. The increased porosity offers advantages such as improved permeability and enhanced surface area, which are beneficial for various applications, including filtration and separation processes. Further characterization and analysis are required to elucidate the specific mechanisms and interactions between TiO_2_ nanoparticles and the polymer matrix to optimize and tailor the porous membrane structure for specific applications. The contact angle of the DLHF membrane, which indicates its hydrophilicity, exhibited a gradual decrease with increasing TiO_2_ content in the SPPSu/PPSu DLHF inner surface. This can be attributed to the hydrophilic nature of TiO_2_, which is attributed to the presence of hydroxyl groups on its surface. As the amount of TiO_2_ increased, more hydroxyl groups were available to interact with the surrounding water molecules, leading to a decrease in the contact angle and an increase in hydrophilicity. However, it should be noted that the hydrophilicity of the membrane decreased when the TiO_2_ content exceeded 1.0 wt%. This can be explained by the increased roughness and irregular positioning of the TiO_2_ particles within the membrane structure. These factors hindered the exposure of hydroxyl groups on the surface of the membrane, resulting in a reduction in hydrophilicity. In summary, the addition of TiO_2_ nanoparticles to the SPPSu/PPSu nanocomposite DLHF membranes enhanced their overall porosity and hydrophilicity. The increased porosity observed in the fiber substructure can be attributed to the greater void volume present within it. Porosity refers to the percentage of void spaces or empty areas within a material, and it directly affects properties such as permeability, mechanical strength, and surface area. In the case of the fiber substructure, the presence of more void spaces indicates a higher porosity level. This could be a result of various factors, including the arrangement and packing of fibers, interstitial gaps between fibers, or the incorporation of porogens or sacrificial materials during the fabrication process. The increased void volume contributes to the overall porosity, allowing for enhanced fluid flow, increased surface area for adsorption or reaction, and potentially improved mechanical flexibility. Understanding and controlling the void volume within the fiber substructure are crucial for tailoring the material’s properties to specific applications.

Furthermore, the results obtained for the MWCO (Figure 5b) clearly illustrate a substantial improvement in pore characteristics following the incorporation of TiO_2_ into the inner surface of the SPPSu/PPSu DLHF membrane. The membrane comprising SPPSu/PPSu exhibits an MWCO of approximately 1000 Da. However, with the inclusion of SPPSu-TiO_2 (2.0 wt%)_/PPSu, SPPSu-TiO_2 (1.0 wt%)_/PPSu, and SPPSu-TiO_2 (0.50 wt%)_/PPSu, the MWCO is notably reduced to a range between 300 and 600 Da. This modification of the membrane’s pore structure holds immense significance as it directly influences the membrane’s performance concerning solute rejection and flux.

### 3.4. Thermal Stability of the Membranes

**Figure 6:**
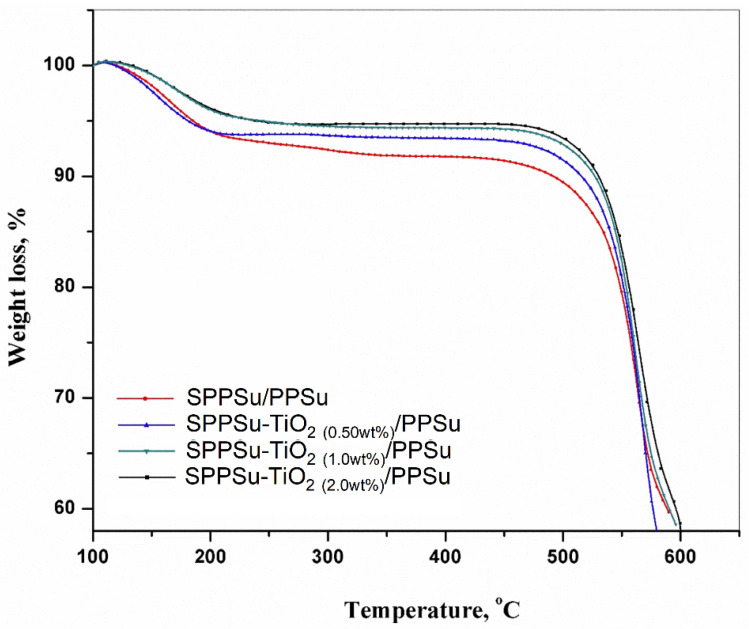
Thermogravimetric analysis of the prepared DLHF membrane.

The thermal stability of the prepared DLHF membrane was analyzed using Thermogravimetric analysis (TGA). TGA curves for both the SPPSu/PPSu membrane and the TiO_2_-added SPPSu/PPSu nanocomposite membrane were obtained and are presented in Figure 6. These curves provide insights into the behavior of the membranes under different temperature conditions ranging from 100 to 600 °C. The incorporation of TiO_2_ nanoparticles into the membrane led to an enhancement in its thermal resistance. The TGA curves of both the pristine and nanocomposite membranes exhibited three primary degradation steps. In the first degradation step, which occurred between 100 °C and 200 °C, a weight loss was observed. This initial weight loss was attributed to the volatilization of volatile components or the evaporation of residual absorbed water. It indicates the removal of any moisture or volatile matter present in the membranes. The second stage of degradation occurred between 200 °C and 550 °C. In this range, a higher weight loss was observed compared to the first stage. This weight loss was primarily attributed to the degradation of the polymeric chain in the membranes. As the temperature increased, the polymeric chains started to break down, resulting in a significant weight loss. Beyond 550 °C, the polymeric chains were completely broken, and the formation of ash began. This stage represents the carbonization of the degraded products, leading to the formation of ash residues. From the TGA analysis, it was concluded that the nanocomposite membrane with TiO_2_ nanoparticles exhibited higher thermal stability compared to the pristine SPPSu/PPSu membrane. The incorporation of TiO_2_ nanoparticles into the DLHF structure enhanced the thermal resistance of the nanocomposite membrane, as evidenced by its ability to withstand higher temperatures without significant weight loss or degradation. Overall, the TGA study provided valuable information about the thermal stability of the prepared DLHF membranes. The presence of TiO_2_ nanoparticles improved the membrane’s resistance to thermal degradation, indicating its potential for applications where high-temperature stability is required.

### 3.5. Mechanical Properties of the Membranes

**Figure 7:**
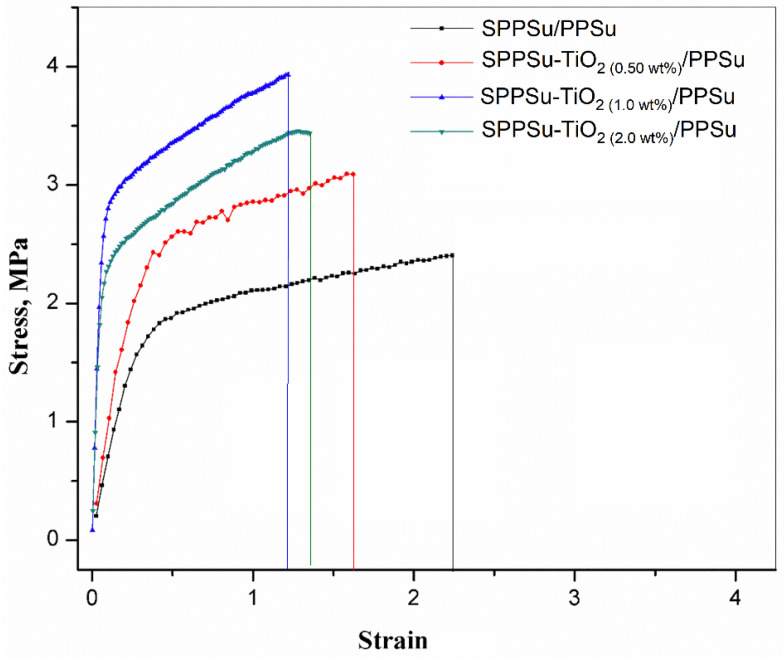
Mechanical properties of the prepared DLHF membranes.

The mechanical properties of the prepared DLHF membrane were investigated and are depicted in Figure 7. The relationship between tensile stress and tensile strain was examined to evaluate the membrane’s performance under mechanical load. A high tensile modulus indicates that the membrane possesses high rigidity. This means that it can resist deformation and maintain its shape under applied stress. The combination of high tensile stress and strain at break is an important characteristic of toughness in materials. It suggests that the membrane can endure significant stretching before reaching its breaking point, indicating enhanced durability. The introduction of TiO_2_ nanoparticles into the DLHF membrane played a crucial role in modifying its mechanical properties. The presence of these nanoparticles resulted in an improvement in the stress values at break. In other words, the membrane became stronger and more resistant to fracture due to the inclusion of TiO_2_ nanoparticles in the fiber membranes. By adding 0.5 to 2 wt% of nanoparticles in the inner layer of the nanocomposite hollow fiber, the tensile stress increased, while the strain decreased. This indicates that the addition of nanoparticles to the SPPSu/PPSu composite material enhances the tensile strength of the membranes. The stress values of the membranes are as follows: SPPSu/PPSu (2.4 MPa), SPPSu-TiO_2_ (0.5 wt%)/PPSu (3.1 MPa), SPPSu-TiO_2_ (1.0 wt%)/PPSu (3.9 MPa), and SPPSu-TiO_2_ (2.0 wt%)/PPSu (3.4 MPa). These values indicate that the addition of TiO_2_ nanoparticles led to a significant increase in the tensile stress compared to the pristine SPPSu/PPSu membrane. It is important to note that the concentration of nanoparticles influenced the mechanical properties. Specifically, the addition of 0.5 to 1.0 wt% of TiO_2_ nanoparticles resulted in improved mechanical properties, while the addition of 2.0 wt% did not yield further improvements. This suggests that an optimal concentration of 1.0 wt% was sufficient to enhance the mechanical performance of the nanocomposite membrane. The mechanism behind the mechanical enhancement of SPPSu-TiO_2_/PPSu nanocomposite membranes by TiO_2_ nanoparticles involves several key factors. First, the addition of TiO_2_ nanoparticles provides a reinforcing effect on the membrane matrix. The nanoparticles act as fillers and create a network structure within the polymer matrix, improving its mechanical strength and rigidity. The presence of TiO_2_ nanoparticles enhances the interfacial interaction between the nanoparticles and the polymer, leading to improved load transfer and stress distribution throughout the membrane. In conclusion, the incorporation of TiO_2_ nanoparticles into the DLHF membrane structure resulted in improved mechanical properties. The nanocomposite membranes exhibited higher tensile stress and increased toughness compared to the pristine membrane. These findings highlight the potential of using TiO_2_ nanoparticles as a means to enhance the mechanical performance of composite membranes.

### 3.6. Performance of Membrane

**Figure 8:**
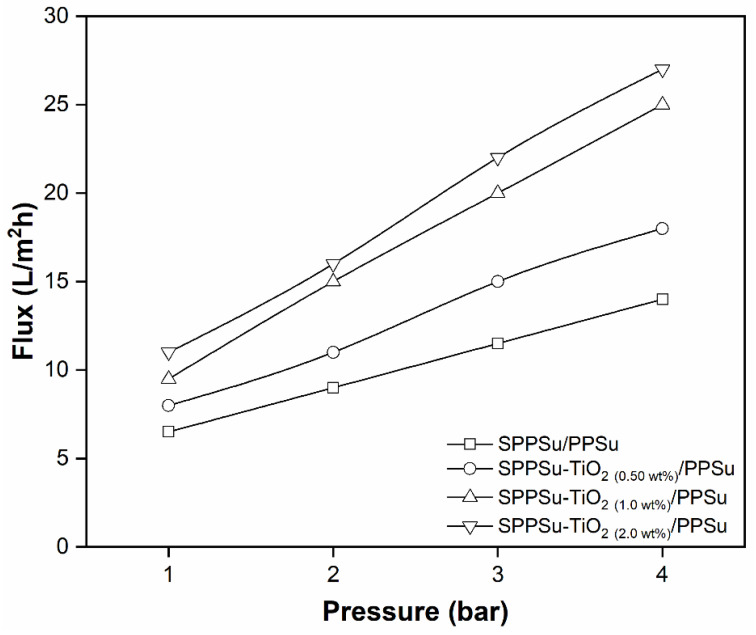
Water permeability of prepared DLHF membranes.

The experimental evaluation of membrane performance in water permeation using a cross-flow set-up revealed significant improvements in the pure water fluxes of DLHF nanocomposite membranes compared to the pure membrane. The water permeability values for the different DLHF membranes were obtained as follows: SPPSu/PPSu (2.5 L/m^2^h.bar), SPPSu-TiO_2_ (0.50 wt%)/PPSu (3.5 L/m^2^h.bar), SPPSu-TiO_2_ (1.0 wt%)/PPSu (5.1 L/m^2^h.bar), and SPPSu-TiO_2_ (2.0 wt%)/PPSu (5.4 L/m^2^h.bar). These results, presented in Figure 8, clearly demonstrated that the incorporation of TiO_2_ nanoparticles into the inner-layer of the SPPSu polymer matrix led to a substantial enhancement in water permeability. The observed increase in water permeability can be attributed to multiple factors. Firstly, the addition of TiO_2_ nanoparticles influenced the pure water flux, resulting in improved water permeability. TiO_2_ nanoparticles have a high affinity for water molecules, leading to increased hydrophilicity of the membrane surface. The enhanced hydrophilicity facilitated the passage of water molecules through the membrane, thereby boosting water permeability. Furthermore, the effects of TiO_2_ nanoparticle incorporation on the membrane morphology played a crucial role in determining the permeation properties. The addition of nanoparticles altered the membrane structure and resulted in changes in pore size, porosity, and surface roughness. These modifications could create more favorable conditions for water transport, contributing to the increased water permeability observed in the nanocomposite membranes. The findings of this study shed light on the design and optimization of DLHF membranes for efficient water permeation applications. Future research can explore the potential of other nanomaterials as additives to further enhance membrane performance.

**Figure 9:**
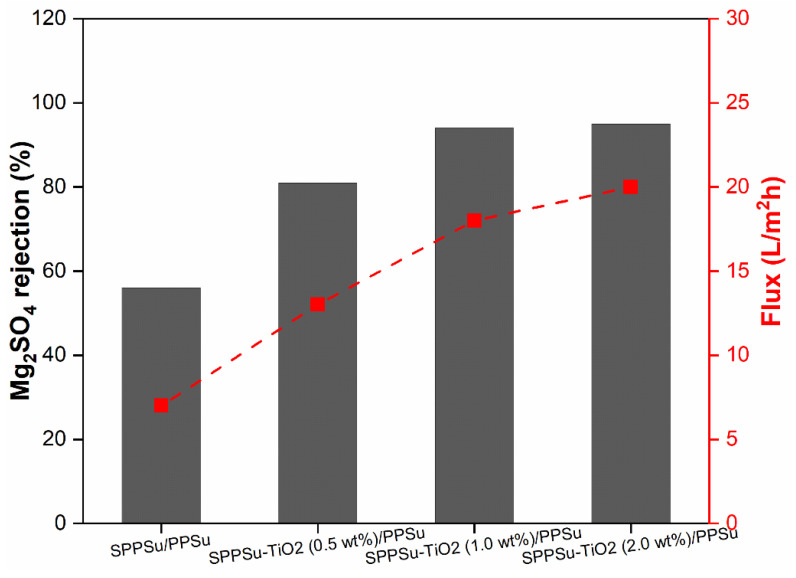
Mg_2_SO_4_ rejection and flux performance of prepared DLHF membranes.

**Figure 10:**
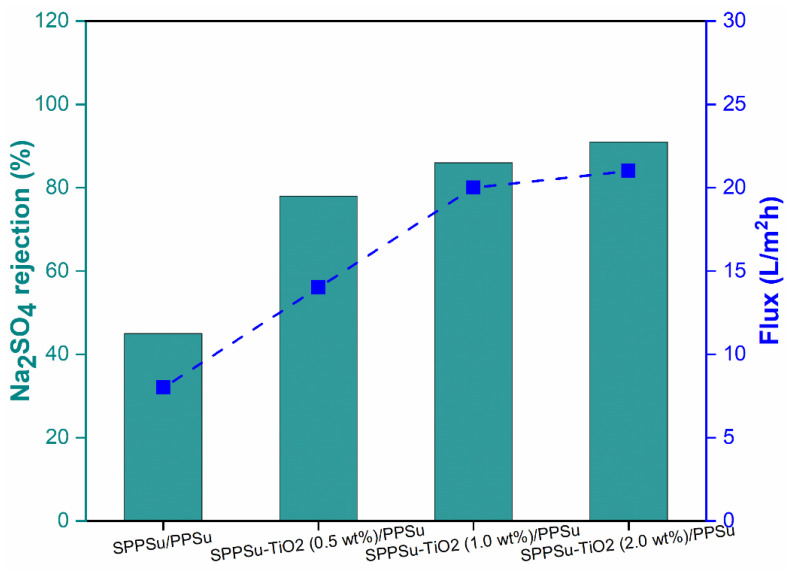
Na_2_SO_4_ rejection and flux performance of prepared DLHF membranes.

The rejection performance of prepared pure SPPSu/PPSu and SPPSu-TiO_2_/PPSu nanocomposite DLHF membranes was evaluated using Na_2_SO_4_ and MgSO_4_ as model salts. A simulated salt solution with a concentration of 500 ppm and a neutral pH was employed as the feed solution. The rejection results are depicted in Figure 9 and Figure 10. The incorporation of TiO_2_ in the inner layer of SPPSu/PPSu nanocomposite DLHF membranes exhibited higher rejection compared to the pure SPPSu/PPSu DLHF membranes. The rejection percentages for MgSO_4_ and Na_2_SO_4_ salts for the different membrane compositions are as follows: SPPSu/PPSu: MgSO_4_ rejection-56%, Na_2_SO_4_ rejection-45%, SPPSu-TiO_2 (0.50 wt%)_/PPSu: MgSO_4_ rejection-81%, Na_2_SO_4_ rejection-78%, SPPSu-TiO_2 (1.0 wt%)_/PPSu: MgSO_4_ rejection-94%, Na_2_SO_4_ rejection-86%, and SPPSu-TiO_2 (2.0 wt%)_/PPSu: MgSO_4_ rejection-95%, Na_2_SO_4_ rejection-91%, respectively. The statistical analysis comparing the present works with other reported studies on salt rejection by hollow fiber membranes reveals notable variations in performance. Liang et al. [38] presented polymeric thin film composite hollow fiber membranes with a commendable NaCl rejection of about 98%, while Ohkame et al.’s [39] hollow-fiber reverse osmosis membrane demonstrated slightly higher performance with a NaCl rejection of 98.3%. Conversely, Zhang et al.’s [40] poly(vinylidene fluoride) hollow fiber membranes exhibited lower salt rejection, measuring below 8.5%. Ian et al.’s [41] study stood out with an impressive NaCl salt rejection of 99.1%. The present results clearly indicate that the incorporation of TiO_2_ nanoparticles in the inner layer of SPPSu/PPSu nanocomposite DLHF membranes significantly enhances the rejection performance for both MgSO_4_ and Na_2_SO_4_ salts. The rejection percentages show a noticeable increase with an increase in TiO_2_ content. This enhancement can be attributed to several factors, including the unique properties of TiO_2_ nanoparticles and the modified membrane structure.

Figure 9 and Figure 10 showed results of Mg_2_SO_4_ and Na_2_SO_4_ rejections for the prepared DLHF membranes. The rejection mechanism of the nanocomposite DLHF membranes can be explained by several phenomena. Firstly, the presence of TiO_2_ nanoparticles in the inner layer introduces additional tortuous pathways for solute transport, resulting in increased diffusion path lengths and improved rejection. This can be attributed to the increased surface area of the membranes and the resulting longer diffusion paths, which hinder the passage of salt ions. Secondly, the TiO_2_ nanoparticles may contribute to the formation of a more compact and dense membrane structure, leading to reduced pore size and increased selectivity. The enhanced rejection can also be attributed to the electrostatic interactions between the TiO_2_ nanoparticles and the salt ions, as TiO_2_ is known to possess surface charges that can facilitate the rejection of charged species. Furthermore, the nanocomposite DLHF membranes demonstrated comparable permeation flux to that of pure water flux, indicating minimal flux decline due to the incorporation of TiO_2_ nanoparticles. This suggests that the presence of TiO_2_ in the nanocomposite membranes does not significantly impede water permeation, indicating their potential for practical applications in water treatment processes. Overall, the incorporation of TiO_2_ nanoparticles in the inner layer of SPPSu/PPSu nanocomposite DLHF membranes leads to improved rejection performance for both Na_2_SO_4_ and MgSO_4_ salts. The rejection mechanism involves increased diffusion path lengths, modified membrane structure, and potential electrostatic interactions. The nanocomposite membranes exhibit high rejection while maintaining permeation flux comparable to pure water, highlighting their potential for water treatment applications. Further research can focus on optimizing the TiO_2_ nanoparticle content and exploring the long-term stability and durability of the nanocomposite DLHF membranes.

## 4. Conclusions

This study investigated the effects of incorporating TiO_2_ nanoparticles into SPPSu/PPSu DLHF. The SEM images of the cross section morphology revealed a moderately symmetric structure in both the inner and outer layers of the membranes, despite the observed delamination between the layers. The addition of TiO_2_ nanoparticles resulted in significant structural changes, including the formation of a thin top layer with nanopores and microvoids in the outer layer. The surface roughness of the inner layer increased with the incorporation of TiO_2_ nanoparticles, indicating a rougher surface. The inclusion of TiO_2_ nanoparticles led to increased overall porosity and hydrophilicity of the DLHF membranes. The addition of TiO_2_ resulted in a more porous membrane structure and a decrease in the contact angle, indicating improved water permeability and enhanced hydrophilicity. However, excessive TiO_2_ content (>1.0 wt%) led to agglomeration and irregular positioning of the nanoparticles, reducing the membrane’s hydrophilicity. The zeta potential measurements showed that the incorporation of TiO_2_ nanoparticles slightly increased the negative charge of the nanocomposite DLHF membranes compared to the pure membrane. This increase in negative zeta potential and the shift in isoelectric point suggest improved fouling resistance for the nanocomposite membranes. The thermal stability of the nanocomposite membranes was enhanced with the incorporation of TiO_2_ nanoparticles, as indicated by the TGA analysis. The nanocomposite membranes exhibited higher tensile stress and increased toughness compared to the pure membrane, demonstrating improved mechanical properties. In terms of performance, the nanocomposite DLHF membranes showed significant improvements in pure water flux compared to the pure membrane. The water permeability increased with the addition of TiO_2_ nanoparticles, attributed to their hydrophilic nature and the modifications in membrane morphology. The rejection performance of the nanocomposite membranes was evaluated using Na_2_SO_4_ and MgSO_4_ as model salts. The incorporation of TiO_2_ nanoparticles in the inner layer of the nanocomposite membranes resulted in higher rejection percentages for both salts compared to the pure membrane. The rejection increased with increasing TiO_2_ content, indicating an improved salt rejection performance.

Overall, the incorporation of TiO_2_ nanoparticles into SPPSu/PPSu DLHF membranes enhanced their structural, surface, thermal, mechanical, water permeability, and rejection properties. These findings provide valuable insights for the development and optimization of DLHF membranes with enhanced performance for various applications, including water treatment and desalination.

## Figures and Tables

**Table 1:** Dual-layer hollow-fiber membrane blend composition.

Membranes	Inner Layer			Outer Layer		
	SPPSu (wt%)	TiO_2_ (wt%)	NMP (wt%)	PPSu (wt%)	PEG 600 (wt%)	NMP (wt%)
SPPSu/PPSu	22	--	78.0	24	2.0	74
SPPSu-TiO_2 (0.50 wt%)_/PPSu	22	0.50	77.5	24	2.0	74
SPPSu-TiO_2 (1.0 wt%)_/PPSu	22	1.0	77.0	24	2.0	74
SPPSu-TiO_2 (2.0 wt%)_/PPSu	22	2.0	76.0	24	2.0	74

**Table 2:** Spinning conditions of dual-layer hollow-fiber membranes.

Parameter	Value
Bore fluid composition (wt%)	Water/NMP(9:1)
External coagulant	Water
Outer dope flow rate (mL/min)	4
Inner dope flow rate (mL/min)	8
Bore fluid flow rate (mL/min)	2
Air gap (cm)	10
Take up speed (m/min)	2
Spinneret temperature (°C)	50

## Data Availability

Not applicable.
